# Enhanced antitumor effects by combining an IL-12/anti-DNA fusion protein with avelumab, an anti-PD-L1 antibody

**DOI:** 10.18632/oncotarget.16137

**Published:** 2017-03-11

**Authors:** Jonathan K. Fallon, Amanda J. Vandeveer, Jeffrey Schlom, John W. Greiner

**Affiliations:** ^1^ Laboratory of Tumor Immunology and Biology, Center for Cancer Research, National Cancer Institute, Bethesda, MD, USA

**Keywords:** checkpoint inhibitor, PD-L1, immunocytokine, interleukin-12, immunotherapy

## Abstract

The combined therapeutic potential of an immunocytokine designed to deliver IL-12 to the necrotic regions of solid tumors with an anti-PD-L1 antibody that disrupts the immunosuppressive PD-1/PD-L1 axis yielded a combinatorial benefit in multiple murine tumor models. The murine version of the immunocytokine, NHS-muIL12, consists of an antibody (NHS76) recognizing DNA/DNA-histone complexes, fused with two molecules of murine IL-12 (NHS-muIL12). By its recognition of exposed DNA, NHS-muIL12 targets IL-12 to the necrotic portions of tumors; it has a longer plasma half-life and better antitumor efficacy against murine tumors than recombinant murine IL-12. It is shown here that NHS-muIL12, in an IFN-γ‒dependent mechanism, upregulates mPD-L1 expression on mouse tumors, which could be construed as an immunosuppressive action. Yet concurrent therapy with NHS-muIL12 and an anti-PD-L1 antibody resulted in additive/synergistic antitumor effects in PD-L1‒expressing subcutaneously transplanted tumors (MC38, MB49) and in an intravesical bladder tumor model (MB49). Antitumor efficacy correlated with (a) with a higher frequency of tumor antigen-specific splenic CD8+ T cells and (b) enhanced T cell activation over a wide range of NHS-muIL12 concentrations. These findings suggest that combining NHS-muIL12 and an anti-PD-L1 antibody enhances T cell activation and T cell effector functions within the tumor microenvironment, significantly improving overall tumor regression. These results should provide the rationale to examine the combination of these agents in clinical studies.

## INTRODUCTION

In prior preclinical studies, the monotherapy antitumor effects of two distinct immunotherapeutic molecules, an immunocytokine, NHS-muIL12, and an anti-programmed cell death protein-1 ligand (PD-L1) antibody, avelumab, have been reported [1, 2]. NHS-muIL12 is a novel immunocytokine delivery system whose antitumor actions are based on two properties. The Ig portion, NHS76 [3], recognizes DNA/DNA-histone complexes exposed within the necrotic portions of tumors, providing a mechanism for selective tumor targeting of IL-12. NHS-muIL12, consisting of the NHS76 antibody fused with 2 molecules of murine IL-12, has been shown to increase serum IFN-γ levels, upregulate MHC class I protein expression on dendritic cells (DCs), and induce the proliferation of CD49b^+^ natural killer (NK) cells and CD8^+^ T cells [1], all consistent with the known properties of recombinant murine IL-12 (rmuIL-12) [4–7]. Moreover, the antitumor effects of NHS-muIL12 were dose-dependent over a 50-fold range and superior to those of recombinant muIL-12 when compared on an equimolar basis. An immune cell subset depletion study confirmed that the antitumor effects of NHS-muIL12 required CD8^+^ T cells and NK cells, again consistent with the known mechanisms of action of IL-12 [1, 6]. Thus, tumor targeting and a blunted *in vivo* bioactivity of NHS-muIL-12 could potentially deliver significant antitumor effects while mitigating some of the dose-limiting toxicity reported in patients treated with rIL-12 [8, 9]. A recently completed first-in-human phase I dose escalation clinical trial of NHS-IL12 demonstrated the agent to be well-tolerated and elicited preliminary evidence of clinical benefit in patients diagnosed with late-stage cancers [10].

The programmed cell death protein-1 (PD-1)/PD-L1 interaction is a well-studied target for immune-based therapy. Blockade of PD-1/PD-L1 binding by the administration of either an anti-PD-1 or anti-PD-L1 antibody overcomes immune resistance in preclinical models [11–17] and has led to remarkable clinical responses in a variety of cancer patients [2, 18–24]. In a previous study, systemic administration of avelumab, a human IgG1 anti-PD-L1 antibody, that recognizes murine PD-L1, was reported to be a highly effective therapeutic agent in the orthotopic bladder tumor model [2].

NHS-IL12 and anti-PD-L1 antibodies approach antitumor immunity from different mechanisms. Targeting IL-12 to the tumor microenvironment results in local IL-12 accumulation that primes a host adaptive immune response. The effectiveness of anti-PD-L1 treatment depends on the release of resident T cells within the tumor microenvironment from the suppressive actions of the PD-1/PD-L1 axis, allowing those T cells to proliferate and exert their cytotoxic effects. With the positive clinical data from studies evaluating immune checkpoint inhibitors (anti-PD-1 and anti-PD-L1) has come the realization that a significant number of patients remain either unresponsive or mildly responsive to those immune-based interventions. Combining NHS-IL12 with an anti-PD-L1 antibody, one would anticipate enrichment of the known immune-related actions of this strong T_H_1 cytokine while blocking the immune suppression within the tumor microenvironment through the interruption of the PD-1/PD-L1 axis. In addition, NHS-muIL12 is rather promiscuous in that it has the ability to be used in combination with radiation, sunitinib, gemcitabine, and docetaxel, resulting in additive and/or synergistic antitumor effects [1, 25]. The present study reports that treatment with an anti-PD-L1 antibody is also an effective monotherapy against subcutaneous as well as intravesical bladder murine tumors in syngeneic hosts. In addition, the results also demonstrate that the combined treatment of NHS-muIL12 and an anti-PD-L1 antibody mediate additive antitumor responses when compared with each monotherapy. Enhanced antitumor efficacy of the combined treatment was associated with a higher number of tumor antigen-specific splenic CD8^+^ T cells. In a separate study [26], the combined treatment of NHS-muIL12 and anti-PD-L1 induced regression of EMT-6 breast tumors. In addition, tumor regression was accompanied with tumor-specific immune memory that protected mice against rechallenge with EMT-6 tumor cells. The combined treatment dose-dependently stimulated cytotoxic NK and CD8+ T cell proliferation and NHS-muIL-12 treatment induced CD8^+^ T cell infiltration into the tumor microenvironment [26]. The results from these two studies provide the rationale for combining an immune checkpoint inhibitor, such as an anti-PD-L1 antibody, with an immunocytokine, NHS-muIL12, as a novel combinatorial approach to immune-based antitumor therapy.

## RESULTS

### Binding of anti-PD-L1 antibodies to murine tumor cells

**Figure 1:**
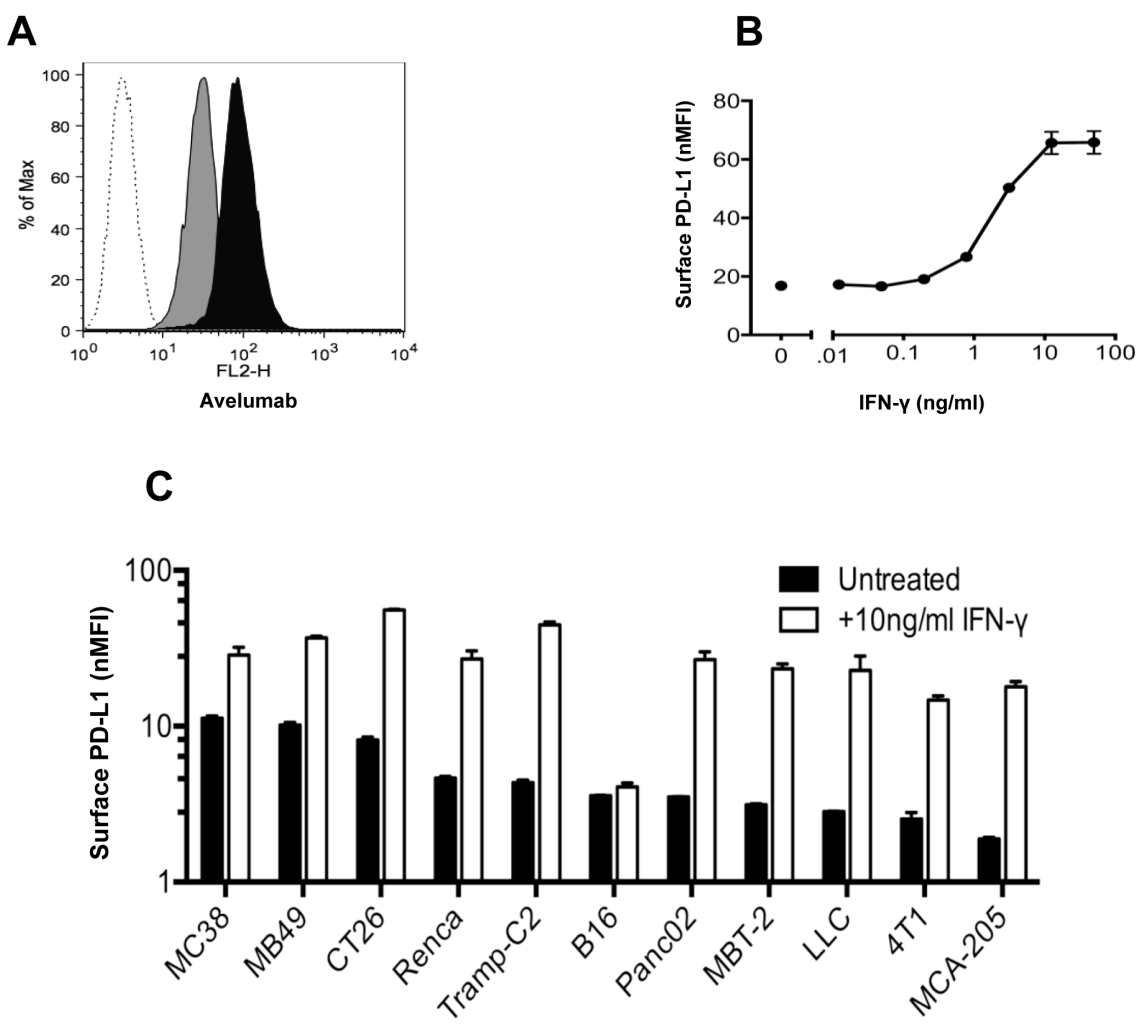
*In vitro* binding of avelumab, a human anti-PD-L1 antibody, to murine tumor cells **A**. MC38 cells were grown *in vitro* for 2 days with or without 10ng/ml IFN-γ. The cells were then labeled with HuIgG_1_ or avelumab (0.2μg/10^6^ cells) and PE-conjugated goat anti-human IgG (0.5μg/10^6^ cells). A representative histogram showing HuIgG_1_ (isotype control) binding to untreated MC38 cells (white histogram) and avelumab binding to untreated MC38 (gray histogram), and IFN-γ-treated MC38 cells (black histogram). **B**. MC38 mouse tumor cells were grown *in vitro* for 2 days in the presence of 0-50ng/ml IFN-γ, and then surface-labeled with avelumab and a secondary antibody. For each condition, the normalized median fluorescence intensity (nMFI) was calculated as the ratio of avelumab binding (MFI) to HuIgG_1_ control binding (MFI) to the tumor cell surface. Circles represent the nMFI +/− SD of duplicate samples for each condition. **C**. Murine tumor cells were grown *in vitro* for 2 days with or without 10ng/ml IFN-γ. For each cell line, the nMFI was calculated as the ratio of avelumab binding (MFI) to HuIgG_1_ control binding (MFI) to the tumor cell surface. Bars represent the nMFI ± SD of duplicate samples for each condition.

Initial studies determined the extent to which avelumab, a human anti-PD-L1 antibody, interacts with mouse PD-L1 (mPD-L1) on different murine tumor cells. Flow cytometric analysis revealed that murine colorectal tumor MC38 cells constitutively expressed mPD-L1 on the cell surface (Figure 1A), which was significantly increased following incubation in the presence of 10ng/ml IFN-γ, as measured by avelumab binding. The increase in mPD-L1 expression levels and subsequent binding of avelumab was dose-dependent across a range of IFN-γ doses (0.01-50ng/ml) (Figure 1B). Avelumab binding to murine MB49, CT26, and LLC cells was also increased in a dose-dependent manner following treatment with IFN-γ (Supplementary Figure S1A-S1C). Cell surface staining with avelumab was used to rank the commonly used murine tumor cell lines based on constitutive mPD-L1 expression. Expression levels varied over a five-fold range from high (MC38, MB49) to low (4T1, MCA-205) mPD-L1-expressing cells (Figure 1C). All 11 cell lines examined had increased mPD-L1 expression levels following IFN-γ treatment, with one exception, B16 melanoma tumor cells. A similar experiment using a commercially available rat anti-PD-L1 antibody confirmed the high-to-low ranking of mPD-L1 expression as well as increased expression following IFN-γ treatment among murine tumor cell lines (Supplementary Figure S1D). In addition, no correlation was seen between IFN-γ‒mediated PD-L1 expression levels and responsiveness to immunotherapy.

### *In vivo* antitumor efficacy of avelumab

**Figure 2:**
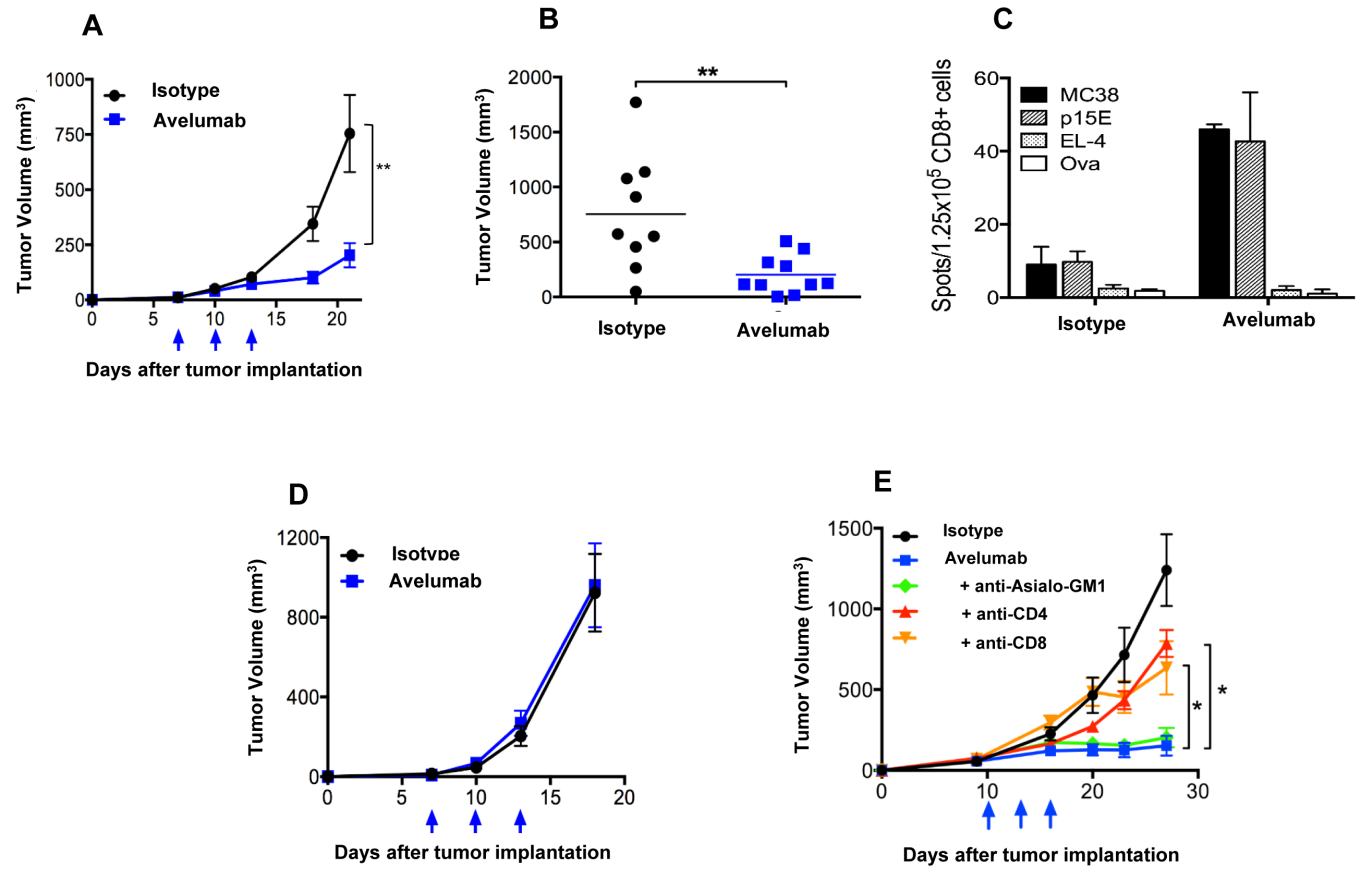
T cell-dependent anti-tumor activity of avelumab on different murine tumor models **A**. and **B**. Immunocompetent C57BL/6 mice bearing subcutaneous (s.c.) MC38 tumors were randomized (*n* = 10/group) and injected i.p. with 400μg avelumab or an isotype control antibody on days 7, 10, and 13. Data representing **A**. mean tumor volume ± SEM over time (arrows indicate treatment days) and **B**. individual tumor volumes on day 21 are shown (bars indicate mean tumor volume). Asterisks indicate statistically significant differences between the isotype control and avelumab-treated groups (*t*-test; **, *P* < 0.01). **C**. C57BL/6 mice were injected with MC38 s.c. tumors and treated as in (A). On day 19 (6 days after the final treatment), isolated CD8a+ T cells (1.25×10^5^/well) from pooled splenocytes (*n* = 5/group) were stimulated *in vitro* with irradiated naïve splenocytes (5×10^5^/well) and antigen for 36 hours in a murine IFN-γ ELISpot plate. Irradiated MC38 tumor cells (25,000/well), irradiated EL-4 tumor cells (25,000/well), p15E peptide (10ng/ml), and ova peptide (10ng/ml) were used. Bar graphs represent the spot counts ± SEM of quadruplicate wells for the indicated antigens. **D**. MC38 s.c. tumor-bearing Rag2^−/−^ mice were randomized (*n* = 9-10/group) and injected i.p. with 400μg avelumab or isotype control antibody on days 7, 10, and 13. Data representing mean tumor volume ± SEM are shown. Arrows indicate days of treatment. **E**. MB49 s.c. tumor-bearing C57BL/6 mice were randomized (*n* = 10/group) and injected i.p. with 3×400μg isotype control antibody alone, 3×400μg avelumab alone, or 3×400μg of avelumab together with antibodies to deplete CD4 cells, CD8 cells, or NK cells. Data represent the mean tumor volume ± SEM over time for each treatment group. Arrows indicate days of avelumab treatment. Asterisks indicate statistically significant differences in mean tumor volume between treatment groups (1-way ANOVA with Bonferroni's post-test; *, *P* < 0.05; **, *P* < 0.01).

The MC38 s.c. tumor model was used to evaluate the *in vivo* anti-tumor effects of avelumab. Avelumab treatment significantly delayed the growth of MC38 tumors in C57BL/6 mice (Figure 2A). The difference in mean tumor volume between avelumab-treated (∼202mm^3^) and isotype control-treated mice (∼755mm^3^) was statistically significant on day 21 (Figure 2B) (*t*-test, *P* < 0.01). The dose of avelumab used (400μg per i.p. injection) was found to be optimal in a separate experiment and the number of treatments was limited to three injections within 1 week, which avoided a neutralizing mouse immunogenic response (data not shown).

Subsequent studies examined the mechanisms by which avelumab reduced MC38 tumor growth. *In vitro* studies ruled out directed inhibition of MC38 tumor cells growth by the human anti-PD-L1 antibody (Supplementary Figure S2). Next, mice bearing MC38 tumors and treated with either avelumab or the appropriate control antibody were analyzed for the presence of antigen specific CD8^+^ T cells using an ELISpot assay. Mice treated with avelumab had an increased frequency of CD8^+^ splenocytes specific for MC38 tumor cells as well as the p15E endogenous retroviral tumor antigen [27], as determined by the number of IFN-γ‒producing spots (Figure 2C). Immune-deficient mouse strains were used to confirm the contributions of the adaptive immune system for the anti-tumor efficacy of avelumab. In Rag2^−/−^ B6 mice (lacking mature B and T cells) bearing MC38 s.c. tumors, administration of avelumab elicited no anti-tumor activity (Figure 2D). Likewise, treatment of SCID mice bearing two separate human s.c. bladder tumor models (T24 and 5637) with avelumab resulted in no change in tumor growth. Finally, in C57BL/6 mice bearing s.c. MB49 bladder tumors, the antitumor effects of avelumab were, once again, abrogated, this time in C57BL/6 mice depleted of CD4^+^ or CD8^+^ T cells (Figure 2E).

### NHS-muIL12 upregulation of murine tumor PD-L1 expression

**Figure 3:**
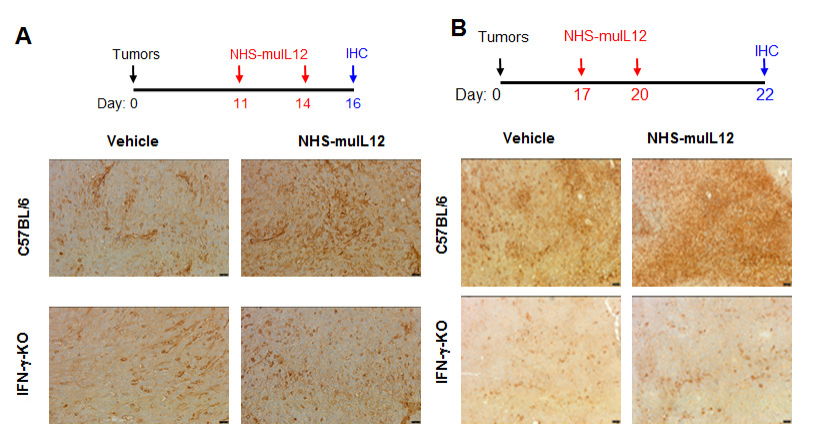
Enhanced PD-L1 expression in MC38 and MB49 s.c **tumors following treatment with NHS-muIL12**. **A**. MC38 s.c. tumor-bearing C57BL/6 mice and IFN-γ-knockout (IFN-γ KO) mice were randomized (*n* = 5/group) and treated with 2μg NHS-muIL12 or vehicle control on days 11 and 14. On day 16, mice were euthanized and tumors were excised, frozen and sectioned for immunohistochemical staining. Frozen tumor sections were labeled with a 1:500 dilution of rat anti-mPD-L1 or rat IgG_2a_ isotype control. Representative images at 200X from one mouse per treatment group are shown. Bar = 25μm. **B**. MB49 s.c. tumor-bearing C57BL/6 mice and IFN-γ KO mice were randomized (*n* = 5/group) and treated with 1μg NHS-muIL12 or vehicle control on days 17 and 20 after tumor implantation. On day 22, the mice were sacrificed and tumors were fixed, sectioned, and labeled with a 1:600 dilution of goat anti-mPD-L1. Representative images at 100X from one mouse in each treatment group are shown. Bar = 50μm.

PD-L1 expression levels on murine tumor cells are highly sensitive and easily upregulated following IFN-γ treatment *in vitro* (Figure 1). Increased serum IFN-γ is one of the immune-based changes that accompany the administration of NHS-muIL12 in mice [2]. Increased serum IFN-γ levels following NHS-muIL12 administration were recapitulated in the present study. Moreover, with the combined treatment of NHS-muIL12 and avelumab, serum IFN-γ levels were further boosted (Supplementary Figure S3). It was of interest to determine to what extent the administration of NHS-muIL12, thereby upregulating serum IFN-γ, affects tumor-associated mPD-L1 expression levels. To address this question, mice bearing MC38 or MB49 s.c. tumors were treated twice s.c. with NHS-muIL12 or an isotype control antibody. Tumors excised from control-treated mice expressed low constitutive mPD-L1 levels, whereas MC38 or MB49 tumors from mice treated with NHS-muIL12 had a clear enhancement of mPD-L1 expression levels (Figure 3A and 3B). The absolute requirement for IFN-γ was established when that same experimental protocol was carried out in IFN-γ-knockout mice: mPD-L1 expression levels remained unchanged following NHS-muIL12 treatment (Figure 3, lower panels in A and B). In a separate study, NHS-muIL12 treatment of BALB/c mice bearing EMT6 breast tumors also increases PD-1 on CD4^+^ and CD8^+^ T cells and PD-L1 expression levels on tumor-associated macrophages, neutrophils and dendritic cells [26].

### Effects of avelumab and NHS-muIL12 on antigen-specific CD8+ T cell activation *in vitro*

**Figure 4:**
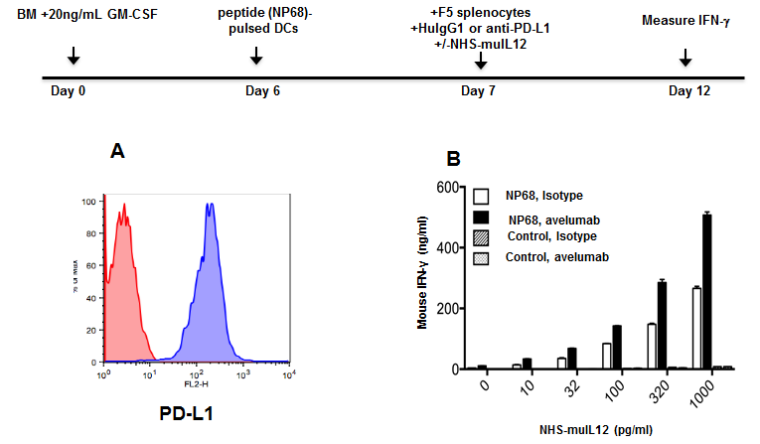
Effects of an avelumab in combination with NHS-muIL12 on T cell activation ***in vitro***. **A**. Bone marrow-derived dendritic cells (BMDCs) were generated *in vitro* by incubating bone marrow cells for 6 days in the presence of 20ng/ml GM-CSF and 10ng/ml IL-4. Non-adherent cells were collected and labeled with HuIgG_1_ control (red histogram) or avelumab (blue histogram) followed by PE-conjugated goat anti-human IgG. **B**. BMDCs (50,000 cells/well) from naïve C57BL/6 mice were pulsed with 100ng/ml NP68 peptide or control peptide overnight in 24-well plates. CD8a+ splenic T cells were isolated from F5 TCR.Tg mice, and added to the plates at 10,000 cells/well. HuIgG_1_ control or avelumab was also added at 10μg/ml, along with 0-1,000 pg/ml NHS-muIL12 in a final volume of 1ml/well. Five days later, supernatant samples from each well were assayed for IFN-γ by sandwich ELISA. Bars indicate mean +/− SE of duplicate wells.

PD-L1 blockade is known to increase the *in vitro* activation of naïve T cells from F5 and OT-I T-cell receptor transgenic (TCR.Tg) mice [2]. IL-12 is well known as one of the more powerful T_H1_ cytokines. It was of interest to examine whether combining NHS-muIL12 with avelumab in an *in vitro* bioassay could predict the effects of that combination on CD8^+^ T cell activation. Bone marrow-derived dendritic cells known to express mPD-L1 were generated from C57BL/6 mice (Figure 4A). Naïve, splenic CD8+ T cells were isolated from F5 TCR.Tg mice and stimulated *in vitro* with the cognate NP68 or control peptide-pulsed BMDCs. The addition of increasing amounts (10-1,000 pg/ml) of NHS-muIL12 in the presence of 10 μg/ml of avelumab to that *in vitro* bioassay boosted IFN-γ production, suggesting additive effects of the combination on antigen-specific T cell activation. No such change in IFN-γ production levels was found, however, when those same T cells were stimulated with a control peptide (Figure 4B).

### Improved antitumor efficacy against s.c. MC38 tumors with the combined treatment of NHS-muIL12 and avelumab

**Figure 5:**
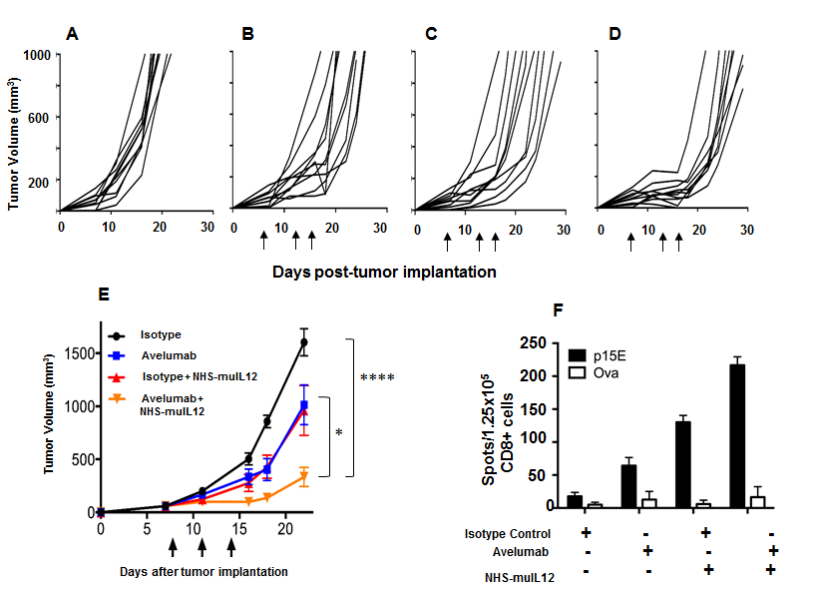
Combined antitumor efficacy of avelumab and NHS-muIL12 ***in vivo***. **A**.-**D**. MC38 s.c. tumor-bearing C57BL/6 mice were randomized (*n* = 10/group) and treated with **A**. 3×400μg HuIgG_1_, **B**. 3×400μg avelumab, **C**. 3×400μg HuIgG_1_ plus 3×2μg NHS-muIL12, or **D**. 3×400μg avelumab plus 3×2μg NHS-muIL12. HuIgG_1_ and avelumab were injected i.p. and NHS-muIL12 was injected s.c. on days 8, 11, and 14 after tumor implantation. As stated previously, NHS-muIL12 injections were limited to three within a week to avoid any neutralization response to the human NHS. **E**. Data represent mean tumor volume ± SEM. Arrows in indicate treatment days. Asterisks indicate statistically significant differences in mean tumor volume between treatment groups (1-way ANOVA with Tukey's post-test; *, *P* < 0.05; ****, *P* < 0.0001). **F**. On day 20, pooled splenic CD8a^+^ T cells (1.25×10^5^/well) from treated mice were stimulated *in vitro* with irradiated splenocytes (5×10^5^/well) and 10μg/ml p15E or ova peptide for 36 hours in a murine IFN-γ ELISpot plate. Bar graphs represent the spot counts ± SEM of quadruplicate wells for each condition.

Treatment of mice bearing MC38 s.c. tumors with either avelumab (400 μg x 3) or NHS-muIL12 (2 μg x 3) were approximately equipotent in delaying tumor growth (Figure 5B and 5C). When combined, however, *in vivo* growth of MC38 s.c. tumor was significantly delayed (Figure 5D). At day 22 post-tumor inoculation, the mean tumor volume in the control-treated mice was ∼1600mm^3^ *versus* ∼334mm^3^ in mice treated with avelumab and NHS-muIL12 (****, *P* < 0.0001; 1-way ANOVA with Tukey's post-test) (Figure 5E). It is interesting to note that combining avelumab at a dose of 400 μg with NHS-muIL12 administered at either a lower (0.5 μg x 3) or a higher (5 μg x 3) dose did not result in additional antitumor effects (data not shown). An ELISpot assay was used to evaluate the magnitude of the T cell response in mice treated with avelumab and NHS-muIL12 alone or in combination. Isolated splenic CD8^+^ T cells were stimulated *in vitro* in the presence of a peptide, a transmembrane component of the retroviral envelope protein p15E, an endogenous component of the MC38 and other murine tumors [27]. Mice treated with the combination of avelumab and NHS-muIL12 had a significantly higher frequency of p15E-specific CD8^+^ splenocytes as compared with those from mice treated with either agent alone (Figure 5F). It should also be noted that mice treated with NHS-muIL12 alone consistently had higher frequencies of p15E-specific splenic CD8^+^ T cells than those treated with avelumab alone.

### Improved antitumor efficacy against MB49 s.c. and I.ves. bladder tumors with the combination of NHS-muIL12 and a rat anti-mPD-L1 antibody

**Figure 6:**
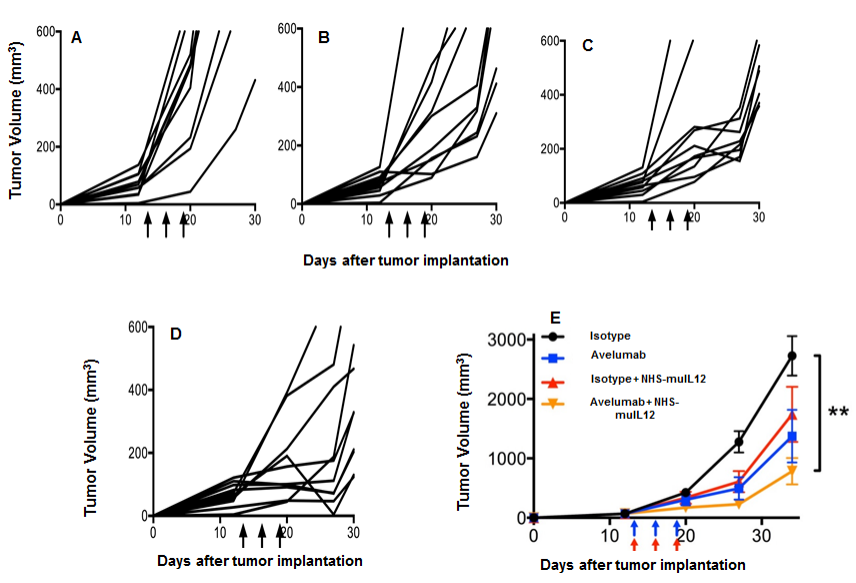
Combined antitumor effects of a avelumab +/− NHS-muIL12 on MB49 tumors (s.c.) MB49 s.c. tumor-bearing C57BL/6 mice were randomized (*n* = 10/group) and treated with **A**. 3×400μg HuIgG_1_ alone, **B**. 3×400μg avelumab, **C**. 3×400μg HuIgG_1_ plus 3×1μg NHS-muIL12, or **D**. 3×400μg avelumab plus 3×1μg NHS-muIL12. HuIgG_1_ and avelumab were injected i.p. and NHS-muIL12 was injected s.c. on days 13, 16, and 19 (arrows). **E**. Data represent mean tumor volume ± SEM over time. Asterisks indicate statistically significant differences between treatment groups (1-way ANOVA with Tukey's post-test; **, *P* < 0.01).

**Figure 7:**
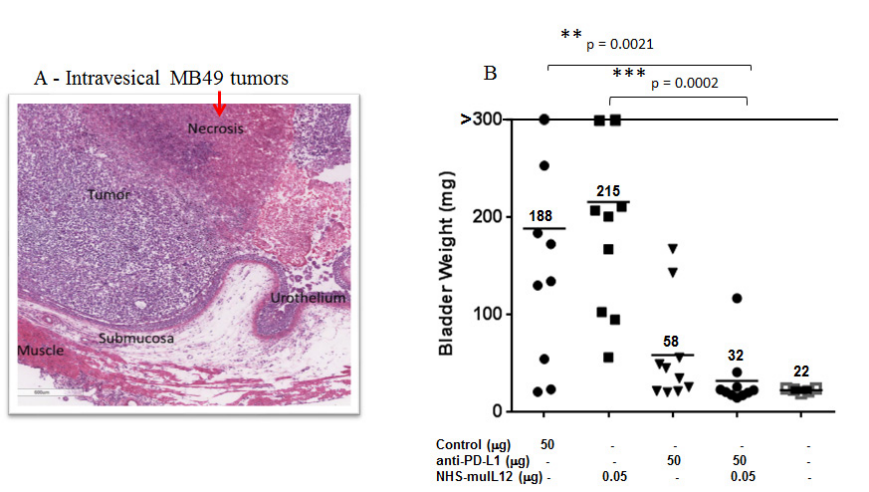
H&E analysis and the combined antitumor effects of a rat anti-mPD-L1 antibody +/− NHS-muIL12 on intravesical bladder tumors **A**. For pathological evaluation, bladders tumors were removed 29 days post-instillation for gross examination and processed by routine histological methods for H&E staining (see Materials and Methods). Magnification images were taken on a Leica DMI4000 microscope with LAS AF (Leica Microsystems Inc., Buffalo Grove, IL). **B**. C57BL/6 mice (n = 10) bearing orthotopic MB49^luc^ bladder tumors were treated as indicated in panel **B**. The rat anti-mPD-L1 antibody was injected i.p. on days 8, 11, 14 and 17, whereas NHS-muIL12 was administered s.c. on days 8, 11 and 14 post-tumor instillation. Mice were euthanized on day 29 and individual bladders were weighed to assess tumor burden. Bladder weights (i.e., 22 mg average) of naïve mice (open squares, no tumor instillation) are also included. Solid black lines indicate the mean bladder weights in each group. **, *P* = 0.0021; control antibody *vs*. anti-PD-L1/NHS-muIL12, ***P = 0.0002; NHS-muIL12 *vs*. anti-PD-L1/NHS-muIL12 (Mann-Whitney test). Data are from a representative experiment.

MB49 murine transitional bladder carcinoma cells form subcutaneous and, when inoculated intravesically, multifocal, non-muscle invasive, non-metastatic bladder tumors. Treatment of subcutaneous MB49 tumors with a human anti-PD-L1 antibody or NHS-muIL12 as monotherapies reduced tumor growth (Figure 6A-6C). Similar to the MC38 tumor model, significant reduction in MB49 tumor growth was achieved only when the two agents were combined (Figure 6D, 6E) (**, *P* < 0.01; 1-way ANOVA with Tukey's post-test). In a separate study, MB49 tumor cells that were instilled into the bladders were found to be highly necrotic (Figure 7A, red arrow). At that time, the mice were imaged, divided into groups and treated with either a rat anti-PD-L1 antibody and/or NHS-muIL12. Dose for the rat anti-PD-L1 antibody was 50 μg (2.5 mg/kg) and for NHS-muIL12 - 0.05 μg (20 μg/kg). On day 29 post-tumor instillation, individual tumor burdens were determined by bladder weights for each treatment group and compared with that of naïve, non-tumor bearing mice (average bladder weight: 22.0 +/− 1.7 mg) (Figure 7B). Mice treated with the isotype-control antibody had considerable tumor burden in that the average bladder weight was more than 8.5-fold that of the naïve, non-tumor bearing mice (188 *vs* 22 mg). NHS-muIL12 administered as a monotherapy at 0.5 μg per injection had no appreciable impact on bladder tumor growth. The rat anti-PD-L1 antibody treatment did inhibit the growth of MB49 bladder tumors as evidenced by the average bladder weight (58 ± 7 mg), but the reduction from mice treated with the control antibody was not significant. Only the combined treatment of the rat anti-PD-L1 antibody and NHS-muIL12 resulted in a statistically significant inhibition of bladder tumor growth [**, *P* = 0.0021; control antibody *vs*. anti-PD-L1/NHS-muIL12, ***P = 0.0002; NHS-muIL12 *vs*. anti-PD-L1/NHS-muIL12 (Mann-Whitney test)].

## DISCUSSION

Antibodies that block the interaction between PD-1 and PD-L1 have recently been approved for the treatment of melanoma, lung, metastatic bladder cancer, advanced renal cell, head and neck and Hodgkin's lymphoma [18–24]. PD-1/PD-L1-blocking antibodies are particularly notable for the long duration of response [19]. Despite the clear utility of these therapeutic agents, most patients show only partial responses or are unresponsive to PD-1/PD-L1 blockade and response rates are especially low for patients whose tumors do not express detectable levels of PD-L1 [19, 22]. Research efforts have expanded to identify other therapies that can be safely combined with anti-PD-1/PD-L1 antibodies in the hope of augmenting clinical utility. The present study (1) establishes the antitumor effectiveness of an anti-PD-L1 antibody in different murine s.c. and intravesical bladder tumor models and (2) provides evidence of heightened therapeutic actions of PD-1/PD-L1 blockade when combined with NHS-muIL12, a pro-inflammatory immunocytokine.

Administration of an anti-PD-L1 antibody and NHS-muIL12 brings different sets of immune-associated actions that coalesce as an effective antitumor combination. The PD-1/PD-L1 axis is associated with suppressing antitumor effects at tumor:T cell interface and by disruption of that axis resident cytotoxic T cells can then exert their control over tumor growth. Studies in Rag2 ^−/−^ mice (Figure 2D) and in mice depleted of CD4 and CD8 T cells (Figure 2E) clearly showed the need for an intact immune system to elicit the antitumor effects of the anti-PD-L1 antibody. PD-L1 is also expressed on antigen-presenting cells (APC). Its binding to PD-1 on T cells recruits phosphatase SHP-2 to the T cell membrane [11, 13], which is reported to inhibit both TCR and CD-28 downstream signaling, thus limiting T cell activation [14, 28]. Disruption of the APC (PD-L1):T cell(PD-1) interaction seems to render T cells more sensitive to antigen (signal 1) and costimulation (signal 2). In the present study, those changes were manifested in the generation of a higher number of tumor antigen specific CD8^+^ T cells (Figure 2C). Delivery of IL-12 *via* a tumor necrosis‒targeting human IgG1 (NHS76) provides an immediate source of a T_H_1 proinflammatory cytokine within the tumor microenvironment. By binding to the IL-12 receptor (IL-12R) on T cells, NHS-muIL12 could provide an additional third signal to direct differentiation along the T_H_1 pathway. IL-12R activation triggers Jak2/Tyk2 signaling, promotes STAT4 phosphorylation, and enhances T_H_1 gene transcription [6]. Another potential source of IL-12 within the tumor microenvironment might come from “newly” activated mature CD8 alpha^+^ DCs which were shown to be enhanced following parenteral administration of NHS-muIL12 [1]. By combining PD-L1 blockade with NHS-muIL12, those complementary signaling pathways for CD8^+^ T cells and NK cells could become fully activated, which would explain the increases seen in the cytotoxic effector functions (Figures 4B and 5F) and, ultimately, in better immunotherapy (Figures 5E, 6E and 7B).

In murine/primate preclinical models and in a recent clinical trial, NHS-IL12 administration enhanced serum IFN-γ production [1], which increased PD-L1 expression on tumors (Figure 3). Since NHS-muIL12 targets the necrotic areas of tumors, the enhanced PD-L1 expression level is probably associated with NK and CD8^+^ T cells in the tumor microenvironment and their propensity for local IFN-γ production. As a monotherapy, NHS-muIL12 does reduce tumor growth, but complete tumor regression occurs only at higher doses (i.e., 5 -10 μg) than used in the present study. The ability of higher doses of NHS-muIL12 to induce complete tumor regression might be easily explained by the higher level of the immunocytokine being targeted to the tumor microenvironment. The administration of NHS-muIL12 at doses ranging from 1-2 μg reduces tumor growth, but the regression is transient probably due to (1) little necrotic areas in s.c. tumors at 7-10 days post-inoculation, thus limiting tumor targeting of the NHS-muIL12, and/or (2) local IFN-γ production that reignites the PD-1/PD-L1 immunosuppressive axis. Similarly, treatment with the anti-PD-L1 antibody activates tumor-associated resident CD8^+^ T cells, but, once again, the reactivation of the PD-1/PD-L1 axis was limiting. Only the combination of NHS-muIL12 with an anti-PD-L1 antibody achieved statistically significant regression of tumor growth. In the intravesical bladder tumor model, the combination of a rat anti-PD-L1 with NHS-muIL12 was more potent and resulted in complete tumor regression. In that model, tumor necrosis occurred early after bladder instillation, which increased NHS-muIL12 targeting to the tumor microenvironment. The increased level of NHS-muIL12 localized to the bladder tumors would be expected to enrich the tumor microenvironment with cytotoxic immune cells. An enhanced accumulation of cytotoxic T cells has also been reported after the combined treatment of mice bearing EMT-6 breast tumors [26]. Such “critical mass” of immune cells, which includes T cells that recognize the endogenous p15E tumor antigen, possibly other neo-antigens as well as NK cells, provides a more robust immunogenic tumor microenvironment that allows the interruption of the PD-1/PD-L1 axis to sustain a more potent antitumor response leading to complete tumor regression. NHS-IL12 is an excellent candidate to combine with an immune checkpoint inhibitor. The ability of NHS-IL12 in the presence of an anti-PD-L1 antibody to (a) induce IFN-γ production, which promotes PD-L1 expression levels, (b) enhance T cell activation *via* changes within the immunological synapse and (c) activate NK cells could contribute to tumor destruction through antibody-dependent cellular cytotoxicity (ADCC) [29]. In the present study, NHS-muIL12 and avelumab were administered simultaneously within 7-9 days, which avoids the onset of neutralizing mouse anti-human antibodies [1]. Subsequent studies should also utilize murine versions of the immunocytokine and anti-PD-L1 and examine the temporal effects of the combined treatments. One possibility is that the antitumor effects might be more effective when the NHS-muIL12 is delivered prior to the anti-PD-L1 checkpoint inhibitor. In any case, both NHS-IL12 and avelumab have been evaluated in separate phase I and II clinical trials [10, 30], and avelumab is currently being evaluated in several Phase 3 studies. The data reported here provide the rationale to potentially evaluate these immune-based therapies in combination.

## MATERIALS AND METHODS

### Animal models and tumor cell lines

Adult female C57BL/6 and Balb/c mice were purchased from Charles River Laboratories (Frederick, MD). IFN-γ^−/−^ (knockout) (C57BL/6 background) mice were purchased from Taconic Biosciences (Rensselear, NY). F5 TCR.Tg mice were purchased from the Jackson Laboratory, Inc. (Bar Harbor, ME). Animals were housed, bred, and maintained in microisolator cages in compliance with *The Guide for Care and Use of Laboratory Animals* (National Research Council).

MC38, MB49, B16, LLC, and MCA-205 cells were grown *in vitro* in DMEM containing 10% fetal bovine serum (FBS), 0.1mM non-essential amino acids (NEAA), 1mM sodium pyruvate, 2mM L-glutamine, 50μg/ml gentamicin, 10mM HEPES, and penicillin/streptomycin. 4T1, CT26, Renca, and MBT-2 cells were maintained *in vitro* in RPMI 1640 medium containing 10% FBS, 2 mM L-glutamine, 50 μM β-mercaptoethanol, 50μg/ml gentamicin, 0.1 mM NEAA, 15 mM HEPES, 1 mM sodium pyruvate, and penicillin/streptomycin. Panc02 cells were grown *in vitro* in McCoy's 5A modified medium containing 10% FBS, 0.1mM NEAA, 1mM sodium pyruvate, 2mM L-glutamine, and 10mM HEPES. Tramp-C2 cells were purchased from ATCC (Manassas, VA) and maintained in the recommended medium.

### Treatments

Two anti-PD-L1 antibodies were used. Avelumab is a fully human, phage display-derived IgG_1_ antibody that binds to both human and murine PD-L1 proteins with high affinity (*K*d = 0.3 and 1.0 nM, respectively). Avelumab and an HuIgG_1_ control antibody were provided by EMD-Serono as part of a Collaborative Research and Development Agreement (CRADA) with the National Cancer Institute (NCI). Both antibodies were produced by transient transfection of mammalian cells *in vitro*, and purified by affinity and ion-exchange chromatography. For *in vivo* studies, tumor-bearing mice were injected intraperitoneally (i.p.) with avelumab or control antibody at a dose of 400 μg in 100 μl PBS per mouse. Mice received three such injections spaced 3 days apart. The second antibody was a rat (IgG2b) anti-mouse PD-L1 (clone 10F.9G2, BioXcell, West Lebanon, NH), which was used for the orthotopic bladder tumor study. In that study, mice received four i.p. injections at 50 μg in 100 μl PBS per mouse. Flow cytometric analyses showed similar binding characteristics for both antibodies on murine tumor and immune cells (data not shown).

NHS-muIL12 is an immunocytokine consisting of a HuIgG_1_ anti-DNA antibody [1, 3] fused to two molecules of the murine IL-12 heterodimer. This immunocytokine was produced by transient transfection of HEK293 cells and purified by Protein A chromatography. For *in vivo* studies, tumor-bearing mice were injected 3x s.c. with NHS-muIL12 at doses ranging from 0.05 to 2.0 μg in 100 μl PBS. NHS-muIL12 and the anti-PD-L1 antibody were administered on the same day.

### Analysis of cell surface mPD-L1 expression

Mouse tumor cells were grown in complete medium for 2 days *in vitro*, with or without recombinant mouse IFN-γ (R&D Systems, Minneapolis, MN). Cells were then trypsinized, washed, and stained with HuIgG_1_ control or a human anti-PD-L1 antibody (0.2μg/10^6^ cells) followed by phycoerythrin (PE)-conjugated goat anti-human IgG (Jackson ImmunoResearch Laboratories, West Grove, PA, 0.5μg/10^6^ cells). Antibody-labeled cells were fixed in BD Cytofix buffer (BD Bioscience Inc., San Jose, CA), and surface PD-L1 levels were detected using a BD FACSCalibur flow cytometer (BD Bioscience Inc). Results were analyzed using the FlowJo software package (FlowJo, LLC, Ashland, OR). Normalized median fluorescence intensity (nMFI) was calculated as: nMFI = human anti-PD-L1 binding (MFI) / HuIgG_1_ binding (MFI).

### Murine tumor models

#### Subcutaneous tumor growth studies

MC38 (3×10^5^) or MB49 (10^5^) tumor cells were injected s.c. into the right rear flank of adult mice. One to 2 weeks later, when the average tumor volume was 40-60 mm^3^, mice were randomized and treatment was initiated. Tumors were measured twice weekly using calipers, and the tumor volume was calculated as: Volume = 0.5 x (width)^2^ x (length).

#### Orthotopic bladder tumors

Intravesical instillation of orthotopic MB49^luc^ bladder tumors was carried out as previously described [31]. Mice were anesthetized (ketamine 15 mg/kg, xylazine 75 mg/kg) and catheterized using Teflon catheters (SurFlash Polyurethane I.V. catheter 24G x 3/4, SR *FF2419, Terumo Medical Products, Somerset, NJ). Orthotopic bladder tumors were established by instilling 7.5×10^4^ MB49^luc^ cells for 45 minutes into bladders that had been pretreated with 100 μL of 0.1 μg/mL poly-L-lysine solution (PLL, MW 70,000 to 150,000) (Sigma-Aldrich, St. Louis, MO). Tumor-take was confirmed by *in vivo* imaging 7-10 days later, at which time mice were placed into groups with equal tumor burden prior to treatment.

### *In vivo* depletion of murine immune cell subsets

In order to selectively deplete CD4+ or CD8+ T cells *in vivo*, mice were injected with GK1.5 or 2.43 antibody, respectively. Mice were injected i.p. with 100μg of the appropriate antibody each day for 4 consecutive days, beginning 1 week prior to tumor implantation. Mice were given additional antibody injections each week throughout the study in order to maintain low T cell numbers. NK cells were depleted by injecting mice weekly with 25μl rabbit anti-asialo-GM1 (Cedarlane Laboratories, Burlington, Ontario, Canada), beginning 1 week prior to tumor implantation and continuing throughout the study.

### *In vitro* generation of dendritic cells from mouse bone marrow

Adult female C57BL/6 mice were euthanized and their leg bone marrow was isolated using a syringe and 26-gauge needle. ACK buffer was used to lyse the red blood cells, and the remaining leukocytes were grown for 6 days in complete RPMI media supplemented with 20ng/ml murine recombinant granulocyte-macrophage colony stimulating factor (GM-CSF) and 10ng/ml murine recombinant IL-4. The media and non-adherent cells were discarded on days 2 and 4 and replaced with fresh media containing GM-CSF/IL-4. On day 6, the non-adherent cells were collected, washed, and used for *in vitro* T cell activation studies. PD-L1 expression on these bone marrow-derived dendritic cells (BMDCs) was determined by cell surface staining with the human anti-PD-L1 antibody and a PE-conjugated secondary antibody. Data was acquired on a BD FACSCalibur flow cytometer and analyzed using the FlowJo software package.

### F5 TCR.Tg T cell activation

After 6 days in culture, BMDCs (50,000/well) were pulsed overnight with 10-1,000ng/ml NP68 peptide (ASNENMDAM, H-2D^b^) or control H-Y peptide (WMHHNMDLI, H-2D^b^) in 24 well-plates. The next day, CD8a+ cells were purified from the spleens of F5 TCR.Tg mice using negative selection magnetic beads (Miltenyi Biotec, San Diego, CA) according to the manufacturer's instructions. Isolated F5 CD8+ cells were added to the 24-well plates at 10,000 cells/well. 10 μg/ml of HuIgG1 or avelumab was also added at this time, together with 0-1,000 pg/ml NHS-muIL12 in a final volume of 1ml/well. After 5 days of *in vitro* T cell activation, supernatant samples were collected and stored at -20°C. Supernatant IFN-γ concentrations were later determined using a standard ELISA kit (Thermo Fisher Scientific, Waltham, MA) according to the manufacturer's instructions. Sample optical densities at 450nm were measured using a Synergy HT plate reader (BioTek, Winooski, VT). Sample IFN-γ concentrations were extrapolated from a standard curve.

### T cell assays (ELISpot)

Splenocytes were pooled for each treatment group, and single-cell suspensions were prepared. CD8a^+^ T cells were isolated by negative selection using magnetic beads (Miltenyi Biotec), then stimulated *in vitro* with irradiated naïve splenocytes and antigen. Specifically, 125,000 isolated T cells were co-incubated with irradiated splenocytes (500,000/well) pulsed with 10μg/ml p15E peptide (KSPWFTTL, H-2K^b,^ ref. [21]), 10μg/ml control VSV-NP peptide (RGYVYQGL, H-2K^b^), irradiated MC38 cells (25,000/well), or irradiated control EL-4 cells (25,000/well). *In vitro* stimulation was carried out in 96 well-plates that had been coated with anti-IFN-γ antibody (Mouse IFN-γ ELISPOT kit, R&D Systems, Minneapolis, MN). After 36 hours, the plate was developed according to the kit manufacturer's instructions. Co-cultures were carried out in quadruplicate. Spot counts were analyzed using an ELISpot plate reader (CTL ImmunoSpot, Shaker Heights, OH) and reported as mean ± SE for each treatment group.

### Immunohistochemical detection

B6 mice bearing either MC38 or MB49 s.c. tumors were administered two s.c. injections of 1-2μg NHS-muIL12 each 3 days apart. Two days after the second injection, tumors were excised, snap-frozen in Tissue-Tek OCT compound (VWR, Inc., Radnor, PA) sectioned, and mounted onto slides. Sections were stained with a 1:500 dilution of rat anti-mPD-L1 antibody (clone MIH5, eBioscience, San Diego, CA) or matched isotype control. All images were acquired with a Leica DMI4000 microscope.

### Serum cytokine quantification

Mouse serum was obtained *via* submandibular bleeding 48 and 72 hours following treatment with NHS-muIL12 and/or avelumab. Murine IFN-γ levels were determined using a standard ELISA kit (Thermo Fisher Scientific) according to the manufacturer's instructions. Sample optical densities (OD) at 450nm were measured using a Synergy HT plate reader (BioTek), and IFN-γ concentrations were extrapolated from a standard curve.

### *In vitro* growth inhibition assay

MC38 cells were incubated for 6 days in complete DMEM in the presence of 0, 0.1, 1.0, or 10 μg/ml HuIgG_1_ control or avelumab. Cells were then trypsinized, washed, and counted using a standard hemocytometer. Each experimental condition was set up in triplicate, and results were reported as fold change ± SE.

### Statistical analysis

For tumor growth studies, the tumor volume over time was plotted as mean ± SE. For single-agent (2-arm) studies, the student's *t*-test was used to analyze the differences in mean tumor volume between treatment groups. For two-agent (4-arm) combination studies, 1-way ANOVA with Tukey's post-test or the Mann-Whitney test was used to analyze the differences in mean tumor volume between treatment groups. GraphPad Prism software (GraphPad Prism 6.01 for Windows, GraphPad Software, Inc., La Jolla, CA) was used for statistical analyses. Differences were significant when the *P* value was less than 0.05.

## SUPPLEMENTARY MATERIALS FIGURES



## Abbreviations

ADCC: antibody-dependent cellular cytotoxicity
APC: antigen-presenting cell
BMDC: bone marrow-derived dendritic cell
CRADA: Collaborative Research and Development Agreement
FBS: fetal bovine serum
GM-CSF: granulocyte-macrophage colony stimulating factor
IFN: interferon
IHC: immunohistochemistry
IL: interleukin
i.p.: intraperitoneally
KO: knockout
MFI: median fluorescence intensity
mPD-L1: mouse programmed cell death protein-1 ligand
NCI: National Cancer Institute
NK: natural killer
nMFI: normalized median fluorescence intensity
OD: optical density
PD-1: programmed cell death protein-1
PD-L1: programmed cell death protein-1 ligand
PE: phycoerythrin
s.c.: subcutaneously
SCID: severe combined immunodeficiency
TCR.Tg: T-cell receptor transgenic
