# Supplementary material for: Enhanced antitumor effects by combining an IL-12/anti-DNA fusion protein with avelumab, an anti-PD-L1 antibody
Source: Oncotarget. 2017 Mar 11;8(13):20558–71. doi: 10.18632/oncotarget.16137 (PMC5400526; doi:10.18632/oncotarget.16137)
Supplement: Supplementary file 1 [file oncotarget-08-20558-s001.pdf]

Enhanced antitumor effects by combining an IL-12/anti-DNA fusion protein with avelumab, an anti-PD-L1 antibody

Supplementary Material

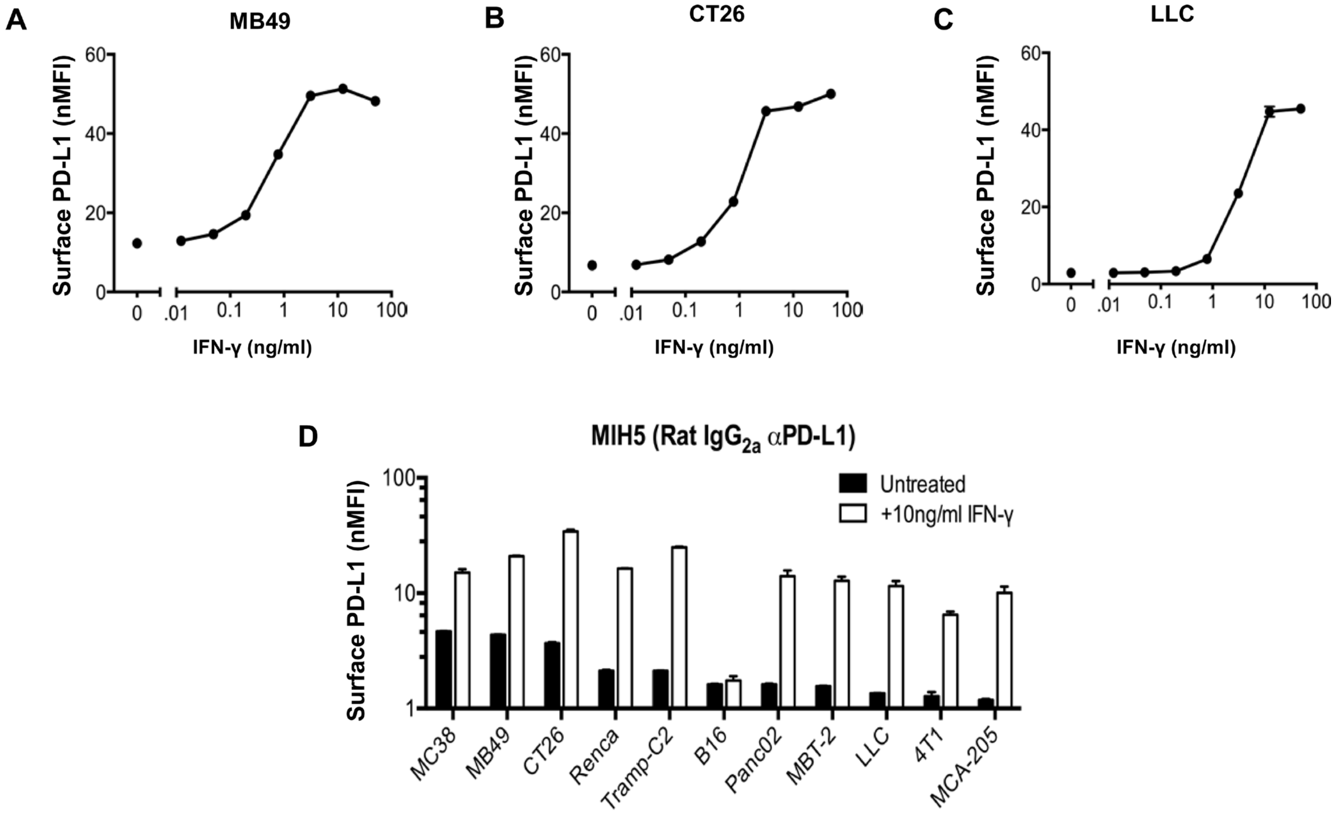

Supplementary Figure S1: Dose-dependent increase in binding of a human anti-PD-L1 antibody to mouse tumor cells following IFN-γ treatment. (A) MB49, (B) CT26, and (C) LLC mouse tumor cells were grown *in vitro* for 2 days in the presence of 0-50 ng/ml IFN-γ. The cells were then surface-labeled with $HuIgG_1$ or a human anti-PD-L1 antibody (0.2mg/10^6 cells) and a PE-conjugated goat anti-human IgG secondary antibody (0.5mg/10^6 cells). (D) Murine tumor cells were grown *in vitro* for 2 days with or without 10ng/ml IFN-γ. For each cell line, the normalized median fluorescence intensity (nMFI) was calculated as the ratio of a human anti-PD-L1 antibody binding (MFI) to $HuIgG_1$ control binding (MFI) to the tumor cell surface. Bars represent the nMFI ±SD of duplicate samples for each condition.

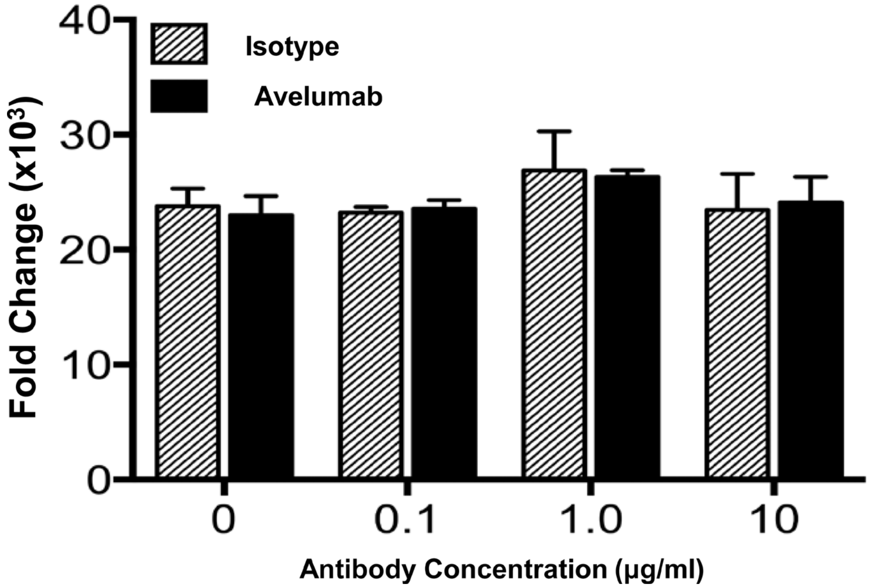

Supplementary Figure S2: *In vitro* tumor cell growth in the presence of a human anti-PD-L1 antibody. MC38 cells were seeded in $75cm^2$ flasks at a density of 500 cells per flask. One day later, $HuIgG_1$ control or a human anti-PD-L1 antibody was added at a concentration of 0, 0.1, 1.0, or 10mg/ml. After 6 days of co-incubation with antibody, the cells were trypsinized, washed, and counted. The fold change was calculated as (final cell count) / (starting cell count). Bar graphs represent the fold change ±SD of triplicate samples.

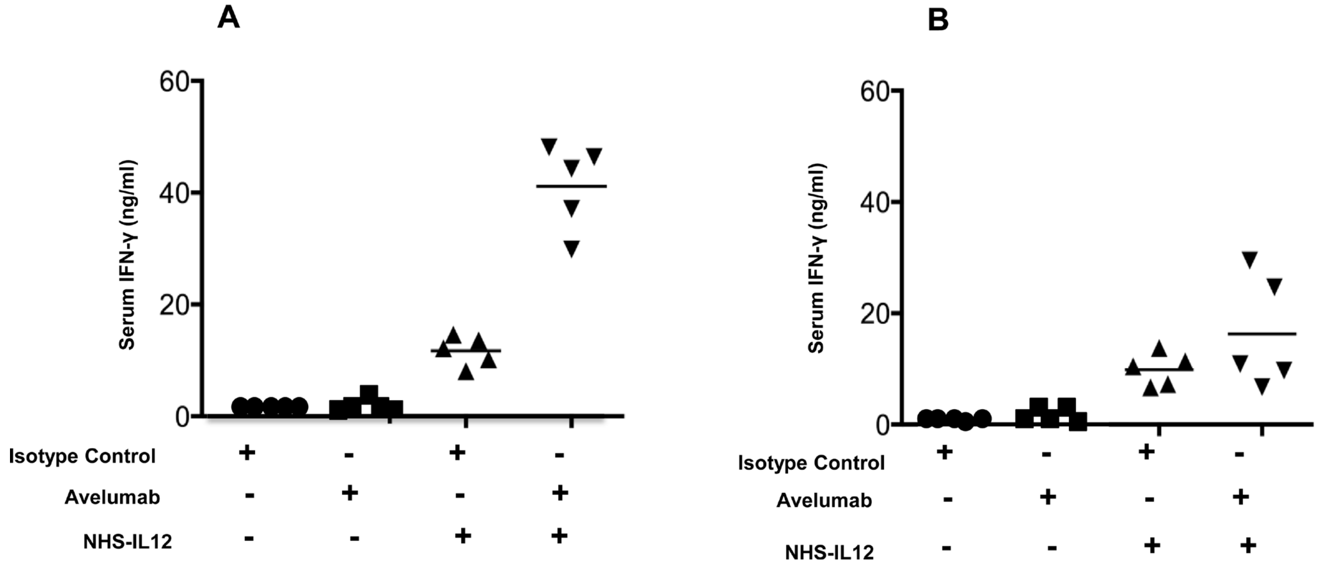

Supplementary Figure S3: Serum IFN-γmeasurement. MC38 s.c. tumor-bearing C57BL/6 mice were randomized (*n*= 10/group) and treated with 3x400mg $HuIgG_1$ alone, 3x400mg human anti-PD-L1, 3x400mg $HuIgG_1$ plus 3x2mg NHS-muIL12, or 3x400mg anti-PD-L1 plus 3x2mg NHS-muIL12 on days 8 and 10 post-tumor inoculation. Serum samples from each mouse were taken 48 hrs (A) and 72 hrs (B) after the last treatment and IFN-γ levels were determined as outlined in the Materials and Methods. Each data point represents a single mouse and the horizontal bar the mean from each of the treatment groups.
